# Impact of Early Electronic Prescribing on Pharmacists’ Clarification Calls in Four Community Pharmacies Located in St John’s, Newfoundland

**DOI:** 10.2196/medinform.3541

**Published:** 2015-01-06

**Authors:** Jennifer L Phillips, Jennifer M Shea, Valerie Leung, Don MacDonald

**Affiliations:** ^1^NL Centre for Health InformationResearch and EvaluationSt John's, NLCanada; ^2^Eastern HealthCancer Care ProgramSt John's, NLCanada; ^3^Canada Health InfowayClinical AdoptionToronto, ONCanada; ^4^Faculty of MedicineMemorial University of Newfoundland and LabradorSt John's, NLCanada

**Keywords:** electronic prescribing, pharmacy, pharmacists, Clinical Pharmacy Information Systems

## Abstract

**Background:**

Electronic prescribing (e-prescribing) can potentially help prevent medication errors. As the use of e-prescribing increases across Canada, understanding the benefits and gaps of early e-prescribing can help inform deployment of future e-prescribing systems.

**Objective:**

The purpose of this exploratory study was to determine the prevalence of, reasons for, and average time taken for pharmacist clarification calls to prescribers for electronic medical record (EMR)-generated and handwritten prescriptions.

**Methods:**

Four community pharmacies in St John’s, Newfoundland, Canada prospectively collected information on clarification calls to prescribers for new prescriptions over a period of 17 to 19 weeks. Four semistructured interviews were conducted following the data collection period to gain further insight.

**Results:**

An estimated 1.33% of handwritten prescriptions required clarification compared with 0.66% of EMR-generated prescriptions. Overall, 1.11% of prescriptions required clarification with the prescriber. While illegibility was eliminated with EMR-generated prescriptions, clarification was still required for missing information (24%) and appropriateness (51%). Key themes, including errors unique to EMR-generated prescriptions, emerged from the qualitative interviews.

**Conclusions:**

Advanced e-prescribing functionality will enable secure transmission of prescriptions from prescribers to a patient’s pharmacy of choice through a provincial electronic Drug Information System (DIS)/Pharmacy Network, which will lessen the need for clarification calls, especially in the domains of missing information and appropriateness of the prescription. This exploratory study provides valuable insight into the benefits and gaps of early e-prescribing. Advanced e-prescribing systems will provide an opportunity for further realization of quality and safety benefits related to medication prescribing.

## Introduction

The National Coordinating Council for Medication Error Reporting and Prevention (NCC MERP) defines medication error as any preventable event that may cause, or lead to, inappropriate medication use or patient harm while the medication is in the control of the health care professional, patient, or consumer, and states that such events may be related to prescribing [1]. The link between medication error and prescribing was further emphasized in 2000, when the Institute for Safe Medication Practices (ISMP) issued a call to action to eliminate the use of handwritten prescriptions, citing this as a source of medication errors. Although only part of a solution to a complex problem, ISMP identified electronic prescribing (e-prescribing) technology to be potentially useful in preventing medication errors [2]. In 2006, the Institute of Medicine recommended that all physicians and pharmacies use electronic prescribing by 2010 [3]. More recently in 2012, the Canadian Medical Association (CMA) and Canadian Pharmacists Association (CPhA) released a joint position that e-prescribing will improve patient care and safety, and committed to a vision that sees e-prescribing in place for Canadians by 2015 [4].

Despite this, there continues to be debate about the impact of e-prescribing technology on patient safety outcomes [5,6]. Previous research has largely focused on the benefits of computerized provider order entry (CPOE) in inpatient hospital environments, while studies of outpatient electronic prescribing have yielded mixed results [7]. Basic computerized prescribing systems with some clinical decision support functionality have been shown to decrease medication error rates in community-based practices [8,9]. However, a study in two US chain grocery stores found no significant difference in the number of pharmacist interventions required when comparing new handwritten prescriptions and electronic prescriptions [10]. It is important to consider the degree of sophistication and system integration of the e-prescribing technology when measuring the value of such systems, as these likely have a substantial impact on the benefits realized [11-14]. In particular, a recent study found that electronic transmission of prescriptions from physicians’ offices to a pharmacy significantly decreased the risk of dispensing errors compared with outpatient CPOE alone [15].

The national landscape of health information technology deployment and adoption are important considerations when contemplating e-prescribing. In Canada, the electronic health record (EHR) is a secure and private lifetime record of a person’s health and health care history, deployed using a series of repository systems. This approach is similar to that used in the United Kingdom, Norway, and the US Department of Veterans Affairs, as well as Kaiser Permanente, a large American health care organization. These repositories collect and store health information in jurisdictionally coordinated repositories. The provincial Drug Information System (DIS) is one component of the EHR. The EHR is then accessed by authorized health care providers using point-of-care systems, such as electronic medical records (EMRs) and pharmacy management systems [16].

E-prescribing is defined as the secure electronic creation and transmission of a prescription between an authorized prescriber and a patient’s pharmacy of choice, using the clinical EMR and pharmacy management software [4]. In Canada, based on the hub-and-spoke model, the DIS component of the EHR serves as a central repository for electronic prescriptions. E-prescriptions would first be transmitted from EMRs to the DIS and then to the pharmacy management system. This is in contrast to the decentralized model traditionally deployed in Scandinavian countries known to be early adopters of e-prescribing, such as Denmark [16,17].

In 2010, Canada Health Infoway commissioned a Pan-Canadian Drug Information Systems study, which included an evaluation of early e-prescribing. At that time, fully evolved e-prescribing was not yet implemented in Canada. Early e-prescribing refers to the use of a stand-alone EMR in a clinician’s office to generate prescriptions (EMR generated) that are printed on paper and then either provided to the patient as they leave, or faxed directly to the pharmacy. Pharmacists surveyed in the study estimated that 40% of prescriptions they received were EMR generated and that these prescriptions required less clarification calls than handwritten scripts [18]. The 2012 Commonwealth Fund survey found that 43% of primary care physicians surveyed used electronic systems for prescribing and, although this increased from 11% in 2006, Canada was still lagging behind other countries in e-prescribing adoption [19]. Results of a recent 2014 national survey of pharmacists reflects similar rates of e-prescribing adoption [20].

Based on this landscape, understanding the attributes of early e-prescribing in Canada will be valuable as fully evolved e-prescribing begins to be deployed across the country. Clarification calls occur when a pharmacist contacts a prescriber to seek clarity on various elements of the prescription and/or to discuss the appropriateness of a medication with the implicit purpose of preventing medication error. Therefore, although clarification calls may only represent one aspect of medication error avoidance, learning more about the frequency and nature of these interventions will help characterize benefits and gaps of early e-prescribing.

The purpose of this study was to explore the prevalence of pharmacist clarification calls to prescribers, the average time taken to perform these clarifications, and how the reasons for clarification calls differ between handwritten and EMR-generated prescriptions at four community pharmacies in St. John’s, Newfoundland and Labrador. At the time of the study, a provincial DIS was being deployed across Newfoundland and Labrador [21].

## Methods

### Recruitment

The research team approached the Pharmacy Network Project Team at the Newfoundland and Labrador Centre for Health Information (the Centre). The Centre is the province’s primary custodian of electronic health data, and is responsible for the development and implementation of the confidential and secure provincial electronic health record, including the provincial Pharmacy Network. The Centre maintains key health databases, prepares and distributes health reports, supports and carries out health analytics and applied health research activities, and undertakes benefits-evaluation projects. Additionally, the Centre provides quality information to health professionals, the public, researchers, and health system decision makers. Given the Pharmacy Network Project Team’s relationship with community pharmacies, they were chosen as a means for recruiting community pharmacies to participate in this study. Four of the five pharmacies identified agreed to participate, while the fifth declined due to work commitments.

### Quantitative Data Collection

Data were collected at the four study pharmacies between July 20 and December 3, 2011, over a period of 17 to 19 weeks, depending on the pharmacy, using a one-page standardized data collection sheet. This data collection sheet was pilot-tested and refined prior to initiation of the study to ensure validity of the tool. Using this tool, pharmacists were asked to document the prescription type received (handwritten or EMR generated), how the prescription arrived at the pharmacy (brought in by the patient or faxed), name of drug(s) prescribed, which drug(s) required clarification, reason for clarification (illegible handwriting, missing information, dose discrepancy from previous prescription, possible drug interaction, allergy, cost contraindication, insurance issue, or other), and time taken for clarification calls to prescribers. Three pharmacies collected data Monday through Saturday. However, to minimize disruption in workflow, the fourth only collected data on alternating days of the week. Only the total number of new prescriptions (no refills) on designated data collection days was recorded. Data were analyzed using SPSS version 17.0.

### Qualitative Data Collection

Four semistructured interviews were completed with participating pharmacists between December 22, 2011 and January 10, 2012. During these interviews, pharmacists were asked about the reasons that prescriptions require intervention, the time spent and methods used for resolving issues, and specific errors frequently associated with handwritten and EMR-generated prescriptions. They were also asked to estimate the percentage of EMR-generated prescriptions received at their practice site. Sessions were audiotaped and transcribed. Interview transcripts underwent thematic/content analysis with the aid of NVivo 9 software. This study was approved by the Health Research Ethics Authority of Memorial University of Newfoundland and Labrador on June 29, 2011 (REB Ref #11.112).

## Results

### Quantitative Data

Overall, for 18,042 new prescriptions filled during the study period, there were 200 (1.11%) clarification calls made. The mean length of time to make a clarification call was 9.1 minutes (SD 5.6). Table 1 provides the characteristics of the study pharmacies, while Table 2 shows the number and percentages of new prescriptions requiring clarification.

An estimated 1.33% (161/12,089) of handwritten prescriptions required a clarification call, compared to 0.66% (39/5953) of EMR-generated prescriptions. For three out of four study pharmacies, the estimated proportion of clarification calls for handwritten prescriptions was higher than that of EMR-generated prescriptions, whereas the fourth pharmacy required clarification for a higher proportion of EMR-generated versus handwritten prescriptions. Across all pharmacies, handwritten prescriptions resulted in the majority of clarification calls to prescribers (161/200, 80.5%).

**Table 1:** Characteristics of study pharmacies.

Pharmacy characteristics	Pharmacy^a^				
	A	B^b^	C	D	Total
Length of data collection time in weeks	18	19	18	17	72
Total number of new prescriptions in study period^c^, n	2539	1980	11,473	2050	18,042
Estimated number^d^of handwritten prescriptions, n (%)	2412 (95.00)	990 (50.00)	7457 (65.00)	1230 (60.00)	12,089 (67.00)
Estimated number^d^of EMR-generated prescriptions, n (%)	127 (5.00)	990 (50.00)	4016 (35.00)	820 (40.00)	5953 (33.00)

^a^The four pharmacies are referred to as A, B, C, and D.

^b^Data for pharmacy B were collected 2-3 days per week over a period of 19 weeks, versus 6 days per week for the other pharmacies.

^c^Verbal (n=2) and missing (n=2) prescriptions were excluded.

^d^Estimates obtained during qualitative interviews with pharmacists.

**Table 2:** Prevalence of clarification calls by prescription type.

Prescription type		Pharmacy^a^				
		A	B^b^	C	D	Total
**All new** ^c^						
	Total, n	2539	1980	11,473	2050	18,042
	Requiring clarification, n (%)	62 (2.44)	61 (3.08)	64 (0.56)	13 (0.63)	200 (1.11)
**Handwritten**						
	Estimated total^d^, n	2412	990	7457	1230	12,089
	Requiring clarification, n (%)	55 (2.28)	43 (4.3)	54 (0.72)	9 (0.73)	161 (1.33)
**EMR generated**						
	Estimated total^d^, n	127	990	4016	820	5953
	Requiring clarification, n (%)	7 (5.5)	18 (1.8)	10 (0.25)	4 (0.5)	39 (0.66)

^a^The four pharmacies are referred to as A, B, C, and D.

^b^Data for pharmacy B were collected 2-3 days per week over a period of 19 weeks, versus 6 days per week for the other pharmacies.

^c^Verbal (n=2) and missing (n=2) prescriptions were excluded.

^d^Estimates obtained during qualitative interviews with pharmacists.

The mean duration of clarification calls for pharmacies A, B, C, and D were 7.2 (SD 4.8), 13.2 (SD 3.1), 7.2 (SD 6.3), and 8.5 (SD 4.6) minutes, respectively. The mean duration of clarification calls for all pharmacies combined was 9.1 (SD 5.6) minutes.

Table 3 shows the reported reasons for clarification calls grouped across four themes: illegibility, missing information (ie, dose, drug, duration, and frequency), appropriateness of the prescription for the patient (ie, dose discrepancy, confirm dosage, known allergy to drug, possible drug interaction, previous adverse reaction, verify directions, and drug), and other (eg, medication not available, insurance, and cost of drug). Some prescriptions had multiple causes for initiation of a clarification call resulting in a total of 236 reported reasons for clarification.

Overall, the most common reason for clarification was to verify the appropriateness of the prescription for the patient (92/236, 39.0%), followed by illegibility (48/236, 20.3%). Dose discrepancy, a reason within the appropriateness category, made up 22.0% (52/236) of all reasons for clarification calls—40 out of 195 (20.5%) for handwritten and 12 out of 41 (29%) for EMR-generated prescriptions. Missing information was the reason for 19.9% (47/236) of calls. In the Other category, the most common reasons were that the medication was not available (15/236, 6.4%)—12 out of 39 (31%) for handwritten and 3 out of 10 (30%) for EMR-generated prescriptions—and insurance issues (12/236, 5.1%)—11 out of 39 (28%) for handwritten and 1 out of 10 (10%) for EMR-generated prescriptions.

**Table 3:** Reasons for clarification calls.

Reason for clarification call	Prescription type requiring clarification call		
	Handwritten (n=195)	EMR generated (n=41)	Total (n=236)
Illegibility, n (%)	48 (24.6)	0 (0)	48 (20.3)
Missing information, n (%)	37 (19.0)	10 (24)	47 (19.9)
Appropriateness of prescription, n (%)	71 (36.4)	21 (51)	92 (39.0)
Other, n (%)	39 (20.0)	10 (24)	49 (20.8)
Total, n	195	41	236

### Qualitative Interviews

Several important themes emerged from the qualitative interviews, with respect to prescriptions requiring intervention and specific errors associated with prescription type. Four themes are reviewed in Textbox 1, including (1) reduction in clarification calls, (2) elimination of illegibility, (3) unique errors with EMR-generated prescriptions, and (4) errors with reprinting prescriptions. When asked about issue resolution, interview subjects cited engaging the patient, using medication profiles, and contacting the prescriber as common interventions. The two pharmacists that were connected to Pharmacy Network noted that while there are limitations due to partial adoption, the Pharmacy Network was beneficial in identifying potential drug abuse and for accessing the patient’s profile. In addition, the medication management program was cited as an enabler for pharmacists to resolve prescription issues independently. Under this standard of practice, pharmacists in Newfoundland and Labrador are authorized to change the form and/or regimen of dosage, change the quantity of medication, complete any missing information, and make nonformulary generic substitutions under specific circumstances, such as having historical information available from a patient’s medication profile [22].

Although illegibility was eliminated, all participants agreed that certain issues with EMR-generated prescriptions still necessitate contacting the prescriber from time to time. For example, participants noted that certain computer-generated defaults, such as quantity calculations and drug catalogs, sometimes lead to error. Interestingly, one pharmacist alluded to incomplete medication reconciliation as a source of missing information: “The doctor will write out a list of medications for somebody and they omit one…and you have to call to get the prescription.” Perpetuation of errors can also be a problem when discrepancies are resolved by the pharmacist at the point of dispensing, but are not documented in the prescriber’s EMR.

**Themes and illustrative quotes from qualitative interviews, with respect to interventions needed and specific errors associated with prescription type.**

Themes with illustrative quotes

1. Pharmacists perceived a reduction in clarification calls with EMR-generated prescriptions compared with handwritten prescriptions.

- “I think the computer-generated ones reduce the amount of calls. There are still calls there but I think there is a reduction.”
- “…if they’re typing it in they might be looking right at their chart because their charts are probably automated as well so that might help…”

2. Illegibility was eliminated with EMR-generated prescriptions.

- “Things that you cannot read are eliminated with computer-generated scripts.”

- “…most times I can’t make out a doctor’s signature, but with computer generated it generates right on the bottom.”

3. EMR-generated prescriptions have errors unique to EMR systems.

- “I find with computer-generated stuff is that they use defaults…little odd things like quantities. I had someone prescribed .6134 of a tablet before because the computer generated something odd.”
- “The computer calculated quantities and a lot of the time the quantities come up as 12.3522…(the clinic next door) is set up for American drugs, too, and we don’t have these here.”

4. Some errors with EMR-generated prescriptions are due to prescribers’ reprinting of prescriptions.

- “I get the same mistakes over and over again with computer-generated slips because they just print them off again every three months…I get errors, the same ones.”

## Discussion

### Principal Results

In this study, the overall prevalence of new prescriptions requiring clarification was 1.11% (200/18,042). At three sites, the estimated proportion of clarification calls for handwritten prescriptions was higher than for EMR-generated prescriptions. The fourth site required clarification for a higher proportion of EMR-generated versus handwritten prescriptions, however, it also had the lowest estimated percentage of EMR-generated prescriptions. Consistent with the literature, an important benefit of EMR-generated prescriptions was elimination of illegibility, however, EMR-generated prescriptions still required pharmacist intervention, mostly due to omission of information and dosing [9,13,14,23]. While clarification calls to prescribers will continue to be required, the need to do so for missing information and dose discrepancies present important opportunities for future benefits possible with advanced e-prescribing.

Whereas missing information and dose discrepancy accounted for 41.9% (99/236) of all reasons for clarification in this study, these actually accounted for 54% (22/41) of reasons for clarifications for EMR-generated prescriptions. Errors unique to EMR-generated prescriptions, such as incorrect rounding or default quantities, were also highlighted by interview subjects. With the average time for a clarification call being 9.1 minutes in this study, a reduction in the need for clarification calls could have a considerable impact on the day-to-day activities of pharmacists. This may also translate to productivity benefits for both prescribers and pharmacists, as previously articulated in a Pan-Canadian DIS study [18].

### Comparison With Prior Work

In the Canadian context, fully evolved e-prescribing will enable secure electronic transmission of prescriptions from prescribers to a patient’s pharmacy via a provincial DIS which may further decrease prescription issues [15]. Connecting EMRs and pharmacy management systems to a DIS necessitates implementation of interoperability standards which may help mitigate issues frequently encountered for EMR-generated prescriptions in this study. For instance, defining mandatory information for successful transmission of a prescription to the DIS may reduce missing information, thereby reducing the need for clarification at the point of dispensing. This is analogous to a “forcing function” designed to prevent omitted information as described by Nanji et al [14]. Similarly, the use of specific terminology for drugs to create alignment between EMR drug catalogs and drug identifiers defined at the DIS level may help reduce prescriptions for products that are not available in Canada. As part of implementation, jurisdictions may also impose business rules that could reduce the likelihood of incorrect default quantities being prescribed, for example, requiring the EMR to display the final prescription to the prescriber for review and validation prior to signing off.

In this study and others, consulting patient medication profiles was a frequent pharmacist intervention for resolving problems with prescriptions [10]. As noted in the interviews, once adoption of the provincial DIS in Newfoundland and Labrador (ie, Pharmacy Network) is complete, a more comprehensive medication profile will become available. This tool may further support pharmacists in assessing the appropriateness of prescriptions by providing relevant context such as titration of dosages over time. Advanced e-prescribing functionality will also enable prescribers to have access to the same medication profile, potentially mitigating issues like unintentional dose discrepancies at the time of prescribing.

Finally, despite issues being resolved at the point of dispensing, it was observed in this study that with EMR-generated prescriptions, errors tend to be repeated upon reprinting. With advanced e-prescribing functionality, information about the final prescription would be recorded in the DIS, not just the local pharmacy management system, decreasing the chance that the identical error could be perpetuated in the future.

### Limitations

This study used an exploratory prospective research design focusing on a small convenience sample of community-based pharmacies in a small Canadian city. The small number of qualitative interviews conducted may also limit generalizability. In addition to a limited sample size, another constraint of this study is that for the purposes of reducing respondent burden, the total number of EMR-generated and handwritten prescriptions was based on pharmacists’ estimates rather than collected prospectively. That said, insights from this small study are important from a lessons-learned perspective. Finally, this study did not quantitatively capture data about pharmacist interventions other than clarification calls, such as discrepancies resolved by the pharmacist using available medication management resources or in collaboration with the patient themselves. Including these may have been useful in understanding how advanced e-prescribing might offer additional benefit in either augmenting or making these types of interventions more efficient.

### Conclusions

This study provides valuable insight around the impact of early e-prescribing on pharmacists’ clarification calls in four community pharmacies located in St. John’s, Newfoundland. While illegibility has been eliminated by computer-generated prescriptions, advanced e-prescribing functionality with connectivity to an electronic provincial DIS provides an opportunity for further realization of benefits related to medication prescribing.

## Abbreviations

CMA: Canadian Medical Association
CPhA: Canadian Pharmacists Association
CPOE: computerized provider order entry
DIS: Drug Information System
EHR: electronic health record
EMR: electronic medical record
ISMP: Institute for Safe Medication Practices
NCC MERP: National Coordinating Council for Medication Error Reporting and Prevention
